# The Dynamics of Mood and Coping in Bipolar Disorder: Longitudinal Investigations of the Inter-Relationship between Affect, Self-Esteem and Response Styles

**DOI:** 10.1371/journal.pone.0062514

**Published:** 2013-04-26

**Authors:** Hana Pavlickova, Filippo Varese, Angela Smith, Inez Myin-Germeys, Oliver H. Turnbull, Richard Emsley, Richard P. Bentall

**Affiliations:** 1 School of Psychology, Bangor University, Bangor, United Kingdom; 2 School of Psychological Sciences, University of Manchester, Manchester, United Kingdom; 3 Greater Manchester West NHS Trust, Manchester, United Kingdom; 4 Department of Psychology and Neuropsychology, University of Maastricht, Maastricht, The Netherlands; 5 Centre for Biostatistics, Institute of Population Health, University of Manchester, Manchester, United Kingdom; 6 Institute of Psychology, Health and Society, University of Liverpool, Liverpool, United Kingdom; Baylor College of Medicine, United States of America

## Abstract

**Background:**

Previous research has suggested that the way bipolar patients respond to depressive mood impacts on the future course of the illness, with rumination prolonging depression and risk-taking possibly triggering hypomania. However, the relationship over time between variables such as mood, self-esteem, and response style to negative affect is complex and has not been directly examined in any previous study – an important limitation, which the present study seeks to address.

**Methods:**

In order to maximize ecological validity, individuals diagnosed with bipolar disorder (N = 48) reported mood, self-esteem and response styles to depression, together with contextual information, up to 60 times over a period of six days, using experience sampling diaries. Entries were cued by quasi-random bleeps from digital watches. Longitudinal multilevel models were estimated, with mood and self-esteem as predictors of subsequent response styles. Similar models were then estimated with response styles as predictors of subsequent mood and self-esteem. Cross-sectional associations of daily-life correlates with symptoms were also examined.

**Results:**

Cross-sectionally, symptoms of depression as well as mania were significantly related to low mood and self-esteem, and their increased fluctuations. Longitudinally, low mood significantly predicted rumination, and engaging in rumination dampened mood at the subsequent time point. Furthermore, high positive mood (marginally) instigated high risk-taking, and in turn engaging in risk-taking resulted in increased positive mood. Adaptive coping (i.e. problem-solving and distraction) was found to be an effective coping style in improving mood and self-esteem.

**Conclusions:**

This study is the first to directly test the relevance of response style theory, originally developed to explain unipolar depression, to understand symptom changes in bipolar disorder patients. The findings show that response styles significantly impact on subsequent mood but some of these effects are modulated by current mood state. Theoretical and clinical implications are discussed.

## Introduction

Attempts to understand the psychological mechanisms underlying bipolar disorder are made difficult by the multidimensional, dynamic and fluctuating nature of the symptoms experienced by patients. For example, although the term ‘bipolar disorder’ implies that depression and mania lie at opposite ends on a spectrum of affect, cross-sectional comparisons indicate that these two groups of symptoms lie on separate dimensions of psychopathology, so that patients can be simultaneously depressed and manic [1], explaining why patients sometimes present with mixed episodes [2]. It has been reported that mood in bipolar patients can fluctuate chaotically over short periods of time [3], and longitudinal studies have shown that, within individuals, manic and depressive symptoms vary relatively independently with each other, although with a small but statistically significant positive correlation between them [4], again explaining why mixed episodes are sometimes observed. The implication of these observations is that psychological studies of bipolar patients should ideally be conducted with sophisticated designs that take into account the complex cross-sectional and longitudinal structure of symptoms, so that covariations between symptoms and psychological processes can be adequately detected.

Problems of self-esteem and related processes seem to be particularly evident in bipolar disorder; almost a century ago, Kreapelin [5] described in detail how manic grandiosity sharply contrasts with low self-esteem and withdrawal during periods of depression. More recent research on the psychological mechanisms in bipolar disorder has focused on self-related cognitive processes already implicated in unipolar depression, for example as proposed in theories by Beck [6] and by Abramson et al. [7]. These studies have shown that individuals with bipolar disorder often present with a negative attributional (explanatory) style [8], a negative self-concept, and dysfunctional attitudes towards the self [9], [10], [11], [12]. In contrast to Kraepelin’s earlier observations, cross-sectional comparisons suggest that these pessimistic cognitive biases may be evident across all phases of bipolar disorder [13].

However, a somewhat different picture has emerged from studies employing longitudinal designs or studies examining symptoms rather than episodes. These studies have indicated that bipolar disorder is associated with substantial instability in affective and self-related processes. Pronounced daily fluctuations in self-esteem have been observed in studies of remitted patients [14], those in depressive episode [13], and also in studies of individuals assessed by questionnaire measures to be at high-risk of bipolar disorder [15]. Further, low self-esteem in persons with bipolar disorder prospectively predicts worsening of affective, particularly depressive, symptoms [10], [16], [17]. In a longitudinal study [18], where patients were assessed every 6 months, although self-esteem correlated positively with current mania and negatively with current depression, negative self-esteem predicted both future depressive and future manic symptoms. Other self-related cognitive measures administered in the study, although correlating with current symptoms, did not predict future symptoms.

In a similar vein, pronounced fluctuations of affect in bipolar disorder have been indicated by studies of high-risk student samples [15], [19], subsyndromal individuals [20], remitted bipolar patients [14], and those currently in manic and depressive episode [13]. Notably, affect and self-esteem appear to fluctuate in concert and hence to be tightly linked [21], [22].

One way of examining shifts in mood and self-esteem is in the context of the coping mechanisms or response styles individuals employ as a response to low, or elevated, mood. In her work on unipolar depression, Nolen-Hoeksema [23] argued that these mechanisms include rumination, problem solving, distraction activities and risk-taking. In a factor-analytic study by Knowles et al. [24], problem-solving and distraction loaded on a single factor they labeled active coping.

A number of studies have found that rumination predicts onset and severity of depression in unipolar patients [25], [26], [27]. Expanding on the original theory, Thomas and Bentall [28] hypothesized that, whilst at times rumination may exacerbate depressive mood in bipolar patients, at other times it may instigate vigorous attempts to avoid negative mood by engaging in high-risk activities resulting, in turn, in hypomania or full-blown mania. Thomas et al. [29] found high levels of rumination in remitted bipolar patients compared to controls, and high levels of self-reported active coping (problem solving and distraction activities) and risk-taking in manic patients compared to controls. Van der Gucht et al. [13] found high levels of rumination in patients in all phases of bipolar disorder, including remission, but again that self-reported risk-taking was elevated only in currently manic patients. Only one study has examined response styles in relation to daily life experiences and fluctuations in mood and self-esteem [15]. In this experience sampling study of high-risk sample of students selected by questionnaire, higher levels of rumination were associated with lower self-esteem, even though no differences in rumination between the low-risk and high-risk groups were identified.

Insight into the temporal dynamics of response styles in relation to other variable psychological processes such as mood and self-esteem has been precluded by the cross-sectional designs employed in most previous studies of bipolar disorder.

Therefore, the aim of the present study was to examine processes specific to bipolar disorder. First, we investigated cross-sectional associations between symptoms of depression and mania with daily life correlates (i.e. affect and self-esteem) and coping styles (rumination, risk-taking and adaptive coping). We predicted that symptoms of depression would be associated with low mood and self-esteem, and more pronounced fluctuations of both. In addition, we expected depressive symptoms to be related to increased levels of rumination. As to symptoms of mania, we predicted associations with increased mood, self-esteem, and their fluctuations. Furhtermore, mania was expected to be associated with risk-taking.

Second, this study sought to examine prospective associations between mood, self-esteem and response styles in two ways: a) whether mood and self-esteem at time T−1 predict engagement in response styles at the subsequent time point. We expected that low mood and self-esteem at time T−1 would predict increased levels of rumination at time T. In turn, high mood and self-esteem would predict increased risk-taking at time T; b) whether engaging in coping styles at time T−1 influences mood and self-esteem at time T. We expected that engaging in rumination would lead to decreased mood and self-esteem, whilst engaging in risk-taking would improve mood and self-esteem.

## Materials and Methods

### Subjects

Ethical approval was obtained from the Leeds (East) Research Ethics Committee and the University of Manchester Senate Ethics Committee. Inclusion criteria for inception into the study were a) diagnosis of bipolar affective disorder, b) currently receiving outpatient care, c) ability to speak/read English, and d) ability to complete the self-report measures independently. Participants were excluded from the study if they met diagnostic criteria for schizophrenia, schizoaffective disorder, primary substance misuse disorder, or had a history of post-natal depression with no hypomania/mania according to DSM-IV [30]. Potential participants were approached via secondary care and self-help groups: 129 covering letters were posted by consultant psychiatrists, resulting in 40 responses, out of which 7 individuals withdrew prior to interview, 5 after receiving further information. Out of the 28 participants commencing the study, 5 dropped out, and 23 completed the study. In addition, consultant psychiatrists approached prospective participants during clinics (N unknown), out of which 3 withdrew after gaining further information, and 24 completed the study. Only one participant was recruited via self-help groups. A total of 48 participants diagnosed with bipolar disorder provided written informed consent and were included into the study: 28 were in a remission, 12 were currently depressed, and 8 currently hypomanic. Participants’ characteristics are described in Table 1. All participants completed the Structured Clinical Interview for Axis I DSM-IV Disorders [31].

**Table 1:** Sample characteristics (N = 48).

Characteristic	Mean (SD) or Percentage
Age (years)	45.42 (10.83)
Age at first episode (years)	27.20 (9.71)
Gender (male/female)	14 (29%)/34 (71%)
Married, cohabiting/Single, divorced or separated	21 (44%)/27 (56%)
Employed/Unemployed/Retired	24 (50%)/18 (38%)/6 (12%)
On antidepressants	65%
On mood stabilizers	71%
On antipsychotics	17%
Remitted	26 (56%)
Hypomanic	8 (17%)
Depressed	12 (26%)
HRSD	4.16 (5.13)
MAS	2.50 (4.26)

Note: HRSD = The Hamilton rating scale for depression; MAS = The Bech-Refaelson mania scale.

### Instruments

#### 1. Clinical measures

To assess symptom levels at the beginning of the study, participants completed two clinical measures in a face-to-face interview.

The Hamilton rating scale for depression [HRSD, 32] consists of 17 items rated by the interviewer on a 0–4 scale with higher scores indicating more sever depressive symptomatology. The HRSD shows inter-rater reliability coefficients up to 0.90 [32], and good validity and reliability [33].

The Bech-Refaelson Mania Scale, Modified Version [MAS, 34] is widely used to assess symptoms of mania and designed to be administered alongside the HRSD. Each of its 11 items is rated on a five-point scale, resulting in a total score ranging between 0–44. The scale shows a high inter-observer reliability and an acceptable level of consistency across items [34].

#### 2. Psychological measures

All variables pertaining to the psychological processes of concern in this study were derived from experience sampling method (ESM) diaries that participants were asked to complete over a six-day period.

Experience sampling method. The experience sampling method (ESM, [35]) is a repeated self-assessment procedure completed in participants’ natural environments and thus advantageous over classically administered self-report questionnaires for its high ecological validity [36]. Its validity, reliability and feasibility have been demonstrated in a number of clinical populations, such as in samples of individuals with diagnosis of schizophrenia [37], [38], depression [39], [40], panic disorder [41] and bipolar disorder [42], [43], [44].

Participants received a pre-programmed digital wristwatch emitting 10 bleeps a day in quasi-random intervals (between 7.30 a.m. and 10.30 p.m.) and six pocketsize diaries to be completed over the period of six days (i.e. one dairy to be completed per each study day). The diary booklet consisted of 10 self-report forms (one per beep), and each comprised scales assessing mood, self-esteem, and styles of coping with depressive mood. Participants received a thorough explanation of the method during a briefing session. To ensure that participants understood the method, they were asked to fill in one form in a trial booklet during the briefing. During the 6-day study period, participants were contacted by telephone to ascertain that they had managed to comply with the procedure, and were thoroughly debriefed after completion of the study. Only participants who completed more than 20 valid responses (i.e. an entry between 5 minutes prior and 15 minutes after the beep) were included in the analyses [45]. This resulted in exclusion of two participants (both females, mean age 59, with depression ratings of 0, 0 and mania ratings of 1 and 2.

### Experience Sampling Method Variables

The items included in the ESM self-assessment forms were all rated on 7-point Likert scales and used to define the following variables:

#### Momentary self-esteem and self-esteem fluctuations

Four items in the self-report form assessed momentary self-esteem (i.e. “I am a failure”, “I am ashamed of myself”, “I like myself”, and “I am a good person”). Using the Kaiser criterion, principal component analysis (PCA) on the raw within-participant scores revealed one factor accounting for 63% of the total variance. Both negative and positive items showed a strong loading on the factor (positive items<−.68; negative items >.80) and high internal consistency after reversing the two negative items scores (Cronbach’s α = .79). The momentary self-esteem score was defined as the mean score of the four items. Each fluctuation in self-esteem was defined as the absolute difference in the ratings of self-esteem between consecutive time points, with higher scores reflecting more intense fluctuations.

#### Positive and negative affect, and mood fluctuations

Nine items assessing momentary positive (e.g. “I feel cheerful”) and negative (e.g. “I feel sad”) affect were used. PCA confirmed two separate factors (eigenvalues >1) together accounting for 66% of variance. The positive affect (PA) factor consisted of four items (“cheerful”, “excited”, “relaxed” and “satisfied”; Cronbach’s α = .82) and the negative affect (NA) factor incorporated five items (“lonely”, “anxious”, “sad”, “irritated” and “guilty”; Cronbach’s α = .86). Fluctuation in mood was defined as the absolute moment-to-moment change in ratings of a) positive mood, and b) negative mood; that is, at each time point two variables were obtained, fluctuation in positive mood and fluctuation in negative mood; higher values reflected more pronounced fluctuations.

#### Assessment of responses to depression

Based on the revised version of Nolen-Hoeksema’s Response Style Questionnaire [23], [24], the self-assessment forms contained eight items evaluating participants’ coping and response strategies for depression (e.g. “Since the last bleep I have thought about the bad things that have happened to me.”) rated on a 7-point Likert scale ranging from −3 (Disagree) to +3 (Agree). Due to bimodal distribution of the scores suggesting that a portion of participants misunderstood the scale as 0 indicating ‘no engagement’, we have recoded all responses rated negatively (i.e. −3, −2, and −1) as 0. Consistent with previous studies [13], [24], PCA confirmed three independent factors accounting for 72% of the variance: rumination (2 items with loadings >.90; Cronbach’s α = .82), adaptive coping (4 distraction and problem-solving items with loadings >.59; Cronbach’s α = .72) and risk-taking (2 items with loadings >.91; Cronbach’s α = .84).

### Data Analyses

The structure of ESM data allows for the investigation of longitudinal associations between ESM variables using regression methods, i.e. testing whether ESM variables at a given beep (i.e. T) are predicted by responses at the previous beep (T−1). The longitudinal nature of these data implies that ESM data have a hierarchical structure (i.e. ESM entries at each beep are clustered within participants); therefore the assumption of the independence of residuals required for linear models is violated. Multilevel modeling adequately account for this type of violations [46], [47], [48]. Data were analyzed with the XTREG module of STATA version 12.0 using maximum likelihood estimation. As a number of variables (i.e. symptoms of depression and mania, and all response styles) were severely positively skewed, bootstrapping (1000 iterations) was utilized, the recommended procedure when the assumptions of normality are violated [49].

Multilevel regression models were employed as follows:

- We investigated the daily life correlates of depressive and manic symptoms measured at baseline. Separate multilevel regression models were estimated for the following dependent variables: PA, NA, SE, fluctuations of PA, fluctuations of NA, fluctuations of SE, rumination, active-copying and risk-taking. For each model, symptoms of depression and mania were entered as independent variables.
- We examined whether PA, NA and SE predicted subsequent response style behaviors. Response style items were phrased “Since the last bleep…” in the diary booklets and as such, assessed coping behaviours between successive time points T−1 and T. For the purpose of the present analyses they were treated as time T items. Separate multilevel regression models were estimated for each independent variable (i.e. PA, NA and SE) as measured at T−1 and response styles (i.e. rumination, active copying and risk-taking) at time T were entered into the models as dependent variables. We controlled for the confounding effect of response style at the previous time point (T−1), as well as for the baseline symptoms of depression and mania.
- We tested whether response styles predicted subsequent levels of PA, NA, and SE. Separate multilevel regression models were estimated for each dependent variable (i.e. PA, NA and SE) at time T with response styles (rumination, adaptive copying, and risk-taking) at time T−1 as predictors. We controlled for the confounding effect of PA, NA and SE at the previous beep, and symptoms of depression and mania measured at a baseline.

## Results

### Are Symptoms of Depression (HRSD) and Mania (MAS) Associated?

In preliminary analyses, we first examined the distributions of depression (HRSD) and mania (MAS) scores, and their associations. As previous studies found a weak, but significant correlation between symptoms of depression and mania [4], [50], we first examined the relatedness of the two scores. Correlation analyses in the present study did not reach statistical significance, r_s_ = 0.18, p = .23. Nevertheless, in the following analyses both symptoms were controlled for simultaneously.

#### i. Are symptoms of depression and mania associated with daily life correlates?

Although our main goal was to investigate the longitudinal relationship between variables, the cross-sectional associations were examined first, see Table 2. First, we investigated whether positive and negative mood, and self-esteem were related to symptom ratings. Statistical analyses were carried out for momentary level of each variable (i.e. PA, NA, SE) as well as their fluctuations. We found that both depression and mania were associated with higher momentary negative affect (p<.001), lower momentary positive affect (p<.001), and lower momentary self-esteem (p<.01), as well as with more pronounced fluctuations of all variables (all *p*s <.001).

**Table 2:** Regression estimates (β) and bias corrected 95% CI for the cross-sectional effects of depression (HRSD) and mania (MAS) on momentary levels of negative (NA) and positive affect (PA) and their fluctuations over time, and on response styles (rumination, adaptive-coping and risk-taking).

Predictor	β(SE)	95% CI	β(SE)	95% CI	β(SE)	95% CI	
	Momentary levels of NA		Fluctuations in NA				
HRSD	.12 (.00)***	[.12.13]	.04(.01)***	[.03.04]			
MAS	.02 (.00) ***	[.01.03]	.01(.00)**	[.00.02]			
	**Momentary levels of PA**		**Fluctuations in PA**				
HRSD	−.12(.00)***	[−.13 −.11]	.01(.00)***	[.01.02]			
MAS	−.02(.01)***	[−.03 −.01]	.01(.00)***	[.01.02]			
	**Momentary levels of SE**		**Fluctuations in SE**				
HRSD	−.12(.00)***	[−.12 −.11]	.03(.00)***	[.02.04]			
MAS	−.01(.00) **	[−.02 −.00]	.02(.00)***	[.01.02]			
	**Rumination**		**Adaptive coping**		**Risk-taking**		
HRSD	.05(.00)***	[.04.06]	.02(.00)***	[.01.02]	.01(.00)***		[.00.01]
MAS	−.01(.00)ns	[−.01.00]	−.00(.00) ns	[−.01.05]	.02(.01)***		[.01.02]

Note: HRSD = The Hamilton rating scale for depression; MAS = The Bech-Refaelson mania scale;

** *p<*.01;

*** *p<*.001*;* ns = not significant.

We also examined the associations between symptom ratings and response style scores (i.e. rumination, adaptive coping, and risk-taking). Depression was significantly associated with higher levels of rumination, adaptive coping and risk-taking (all ps <.001), whilst mania was significantly associated only with increased levels of risk-taking (p<.001; Table 2).

#### ii. Does affect and self-esteem at time T-1 predict response styles at time T?

The main aim of the present study was to examine associations between affect, self-esteem, and response styles over time. We first examined how affect and self-esteem influenced the way individuals engaged in response styles, and then (in the next section), how response styles affected subsequent mood and self-esteem.

First, the predictive properties of each affect and self-esteem variable at each time point (T−1) on rumination at the subsequent time point (T) was investigated (Table 3, upper rows). Multilevel regression analyses revealed that negative affect was associated with increased rumination (p<.001), whereas positive affect (p<.001) and self-esteem (p<.001) were associated with decreases in ruminative thinking at the subsequent time point. When all predictors were entered into the model simultaneously, only affect remained a significant predictor of subsequent rumination: positive affect was associated with a decrease (p<.01), whilst negative affect with an increase (p<.001) of rumination (Table 3 lower rows).

**Table 3:** Regression estimates (β) and bias corrected 95% CI for the longitudinal effect of PA, NA, and SE at time T-1 on response styles at time T.

Predictor	β(SE)	95% CI	β(SE)	95% CI	β(SE)	95% CI
	Rumination		Adaptive coping		Risk-taking	
PA^a^	−0.10(0.02)***	[−.13 −.07]	0.01(0.02) ns	[−.02.04]	0.20(0.08)*	[.03.12]
NA^a^	0.14(0.02) ***	[.10.17]	−0.01(0.02)ns	[−.04.03]	−0.07(0.03)*	[−.13 −.02]
SE^a^	−0.15(0.03)***	[−.19 −.10]	0.01(0.02) ns	[−.02.05]	0.02(0.01) ns	[−.01.05]
PA^b^	−0.05(0.02)*	[−.09 −.01]	0.02(0.01) ns	[−.04.05]	0.03(0.02)¶	[−.00.06]
NA^b^	0.08(0.02)**	[.03.14]	0.01(0.01) ns	[−.03.05]	−0.02(0.02) ns	[−.05.01]
SE^b^	−0.06(0.04)ns	[−.13.01]	0.01(0.02) ns	[−.04.05]	−0.02(0.03) ns	[−.07.03]

Note: PA = positive affect; NA = negative affect; SE = self-esteem;

* *p*<.05,

** *p*<.01

*** = *p*<.001,

ns = non-significant;

a entered into model as a separate predictor;

b entered into model simultaneously.

¶ denotes *p* = .071.

None of the independent variables was significantly associated with adaptive coping (all *p*s = ns; Table 3).

Finally, we examined whether affect and self-esteem at time T−1 predicted risk-taking at time T (Table 3, upper rows). Risk-taking was significantly predicted by high positive (p<.01), and low negative mood (p<.01) at the previous time point, but only positive affect (p = .071) remained marginally associated with risk-taking when all predictors were entered into the model simultaneously (Table 3, lower rows).

#### iii. Do response styles assessed at T-1 predict affect and self-esteem at T?

Multilevel regression models were estimated to examine whether response styles to depression predicted changes in positive affect, negative affect and self-esteem at subsequent time points. When separate models were estimated for a model with positive affect as the dependent variable, adaptive coping (p<.05), and risk taking (p<.01) at the previous time point significantly predicted an increase in positive affect (both *p*s <.05), whilst rumination significantly predicted a decrease in self-esteem, and only marginally in positive affect (p = .05). All predictors were significantly associated with positive affect when entered into the model simultaneously (all *p*s <.05, Table 4).

**Table 4:** Regression estimates (β) and bias corrected 95% CI for the longitudinal effect of rumination, adaptive coping and risk-taking at time T-1 on momentary levels of PA, NA, and SE at time T1.

Predictor	β(SE)	95% CI	β(SE)		95% CI	β(SE)	95% CI
	PA		NA			S-E	
Rumination^a^	−0.07(0.03)¶	[−.14 −.00]	0.05(0.03)ns	[−.01.12]		−0.23(0.03)***	[−.29 −.16]
Adaptive coping^a^	0.10(0.05)*	[.00.19]	−0.03(0.04)ns	[−.11.05]		0.12 (0.03)ns	[−.01.13]
Risk-taking^a^	0.13(0.04)**	[.04.21]	−0.03(0.04)ns	[−.10.04]		0.05(0.04)ns	[−.03.13]
Rumination^b^	−0.10(0.03)**	[−.17 −.03]	0.02(0.03)¶¶	[−.01.12]		−0.05(0.03)ns	[−.11.00]
Adaptive coping^b^	0.12(0.05)*	[.03.22]	−0.05(0.04)ns	[−.13.03]		0.07(0.03)*	[.01.11]
Risk-taking^b^	0.13(0.06)*	[.01.24]	−0.02(0.04)ns	[−10.07]		0.04(0.04)ns	[−.02.11]

Note: PA = positive affect; NA = negative affect; S-E = self-esteem;

* *p*<.05,

** *p*<.01

*** *p*<.001,

ns = non-significant;

a entered into model as a separate predictor;

b entered into model simultaneously.

¶ denotes *p* = 0.050;

¶¶ denotes = .079.

When separate models were estimated with negative affect as the outcome variable, no significant associations were revealed. Nevertheless, in a model with all response styles entered into the model simultaneously, a marginally significant relationship between rumination at time T−1 and negative affect at the subsequent time point was found (p = .079).

In a model with self-esteem as the dependent variable, no significant associations with response styles at the previous time point were revealed. When all predictors were entered into the model simultaneously, adaptive coping at time T-1 significantly predicted an increase in self-esteem at time T (p<.05).

#### iv. Follow-up analyses

In order to examine whether any of the identified relationships were moderated by symptoms of depression or mania, an interaction term between each predictor and symptoms was added into each of the models described in ii) and iii) above with all relevant predictors entered simultaneously. Each model was calculated twice, first with interactions between symptoms of depression and the predictors, followed by a similar model with interactions between symptoms of mania and the predictors. For example, in the case of the model with positive affect as a dependent variable and all three response styles as predictors, three interaction terms were added (between each response style and ratings of depression). A similar model was then calculated with interaction terms between each response style and ratings of mania.

Only one model yielded a significant baseline symptom × predictor interaction. A significant interaction term between symptoms of mania and levels of rumination (β = 0.02, SE = 0.01, p<.01, CI [.01.04]), was found when positive affect was the dependent variable. Additional analyses indicated that rumination led to a decrease in positive affect in individuals with low symptoms of mania at baseline (β = −.27, SE = .04, p<.001, CI [−.35 −.19]) but not in those with high symptoms of mania at baseline. No other significant interaction terms were identified (all *p*s>.05).

## Discussion

The present study is a novel investigation of the prospective relationships between affect, self-esteem and response styles in individuals diagnosed with bipolar disorder. It tests Nolen-Hoeksema’s [23] response style theory and its later adaptations [24], [28], originally formulated to explain the course of unipolar depression using longitudinal data from bipolar patients to examine the impact of psychological variables on response styles and, subsequently, the effect of response styles on psychological variables. The experience sampling method employed in this study allowed the capture of these dynamic relationships, which cannot be assessed using more conventional cross-sectional designs.

Before reviewing the main results, we will comment first on the observed cross-sectional relationships between mood and self-esteem in daily life and baseline symptoms of depression and mania. It was expected that low self-esteem and high negative affect would be associated with symptoms of depression, whereas high positive affect and self-esteem would relate to symptoms of mania. Further, we predicted that increased fluctuations of these processes would be related to both symptoms. Our expectations regarding associations with depression were confirmed, and in line with previous literature. Here, associations between depression and negative mood, as well as its instability, have been consistently reported in studies of high risk students [19], [24], [51], subclinical samples [20] and bipolar patients [13], [52]. Similarly, previous findings have indicated an association between depression and self-esteem [16], as well as instability of self-esteem in high risk student [15] and patient studies [14].

Contrary to our expectations, symptoms of mania showed similar associations with mood and self-esteem as depression (i.e. mania was associated with low mood and self-esteem, and their increased instability), although the effect found was smaller. In contrast to our findings, previous studies have found mania to be related to *high* mood [51], and self-esteem comparable to that of controls [13]. Yet, our findings are not the first of its kind. An earlier factor analytic study suggested dysphoria to be the strongest component of mania [53], and underlying negativity of affect and self-concept during mania have been suggested by studies employing implicit assessments [14], [54].

The discrepancy between the present study and previous reports, both employing explicit assessments, might be related to methodological differences. For example, a number of studies employed comparisons of different phases of bipolar disorder, rather than investigating associations of psychological measures with symptoms (e.g. [13]), an approach complicated by frequent co-existence of depressive and manic symptoms. Another explanation might be related to age differences between examined populations. Several previous studies employed high-risk student populations, and it is likely that personal context of students is considerably different to that of adults with a history of severe mental illness. Although both kinds of studies may be tapping the same underlying vulnerabilities, their expression might be changing across the course of life. The present study is methodologically advantageous in that it has employed patients, representative of bipolar phenomenology, and utilized a longitudinal and ecologically valid assessment and robust statistical methods controlling for covariation of symptoms and non-normality of data.

The increased fluctuations in affect and self-esteem seen in relation to symptoms of depression and mania in the present study suggests that the fluctuations we have observed in remitted patients in previous studies [13], [24] may have been the consequence of subsyndromal symptoms.

In respect of associations between symptoms and response styles, we expected that rumination would be associated with depression, and risk-taking with mania. Indeed, symptoms of depression were related to increased rumination, an observation that is consistent with Nolen-Hoeksema’s [23] original response style theory, and with findings from bipolar high-risk [24], [28], [55], and patient studies [13], [29]. The association observed between depressive symptoms and adaptive coping was unexpected, as an earlier patient study found adaptive coping to be related to mania rather than depression [29]. The disparity might reflect the differences between the retrospective questionnaire assessments employed by Thomas et al. [29] and the more ecologically valid experience sampling method utilized in the current study. Finally, risk-taking was positively associated with symptoms of depression as well as mania. Although we did not predict an association between depression and risk-taking, similar cross-sectional relationships have been reported previously [14], [24], [29].

The main aim of the present study was to examine the unique associations between momentary mood, self-esteem and coping styles, and vice versa, whilst controlling for symptoms of depression and mania. To our knowledge, this is the first study to prospectively investigate Nolen-Hoeksema’s [23] response style hypothesis, utilizing measures of response styles in daily life. It was predicted that both low mood and low self-esteem would prompt rumination at a subsequent time point, whilst positive mood and high self-esteem might trigger risky behaviors. The hypotheses were mostly confirmed, with a number of implications requiring comment. As noted, previous cross-sectional studies reported an association between rumination and symptoms of depression. The present findings suggest that high levels of negative, and low levels of positive affect instigate the subsequent engagement in rumination and that, in turn, rumination impacts most robustly via the dampening of positive mood. Furthermore, rumination led to decrease in positive affect only in individuals with few symptoms of mania, whilst no effect was found in those with manic symptoms. These findings are in line with Nolen-Hoeksema’s notion that rumination as such does not *cause* depression, but rather moderates already depressive mood [56]. The null finding regarding the causal role of self-esteem potentially points to the precedence of affect over cognitive psychological processes in affective disorders, but further investigations are warranted, and this conjecture should be viewed with caution.

The findings regarding risk-taking have both theoretical and clinical implications. Although risk-taking have been found to be related to symptoms of depression and mania cross-sectionally, in a prospective design, positive, rather than negative, mood led to greater risk taking when controlling for the effect of symptoms (although the association reached only marginal significance). In turn, engaging in risk-taking resulted in improvements of mood. In a similar vein, Thomas et al. [29] and Van der Gucht [13] reported higher levels of risk-taking, as measured by questionnaire, in manic participants compared to controls. The failure to detect an association between risk-taking and negative affect, then, implies that this response style might not necessarily act as a defense against low mood as proposed previously [28], but rather is associated with an increased emotional and behavioral reactivity to reward stimuli as proposed by the behavioural activation theory of mania [57], [58], [59]. This account is consistent with recent neuroimaging studies, which have pointed to the abnormal processing of reward stimuli in bipolar patients and at-risk samples [60], [61], [62].

In her original theory, Nolen-Hoeksama (1991) suggested that engaging in distraction (which, along with problem-solving, was incorporated into adaptive coping in this and some previous studies) ameliorates depressive symptoms. Moreover, Nolen-Hoeksema argued that employing healthy coping strategies such as problem solving may be prevented by rumination. Our findings support these hypotheses only partially. Although in the current study neither mood, nor self-esteem instigated subsequent engagement in adaptive coping, employing this coping style led to substantial improvements in mood and self-esteem at the following time point. Furthermore, adaptive coping was found to be an effective strategy even when controlling for other coping strategies. Hence, adaptive coping appears to be a top-down strategy, that can be deliberately employed to improve one’s affective state, an observation that is consistent with earlier studies showing its effectiveness in natural and laboratory conditions [25], [56].

A number of limitations should be acknowledged. Despite methodological advantages of experience sampling method over classical self-report assessments [45], some authors have raised concerns regarding participants’ compliance with, and hence reliability of, the pencil-and-paper protocol of experience sampling, favoring the use of electronic diaries [63], [64], [65]. Whilst this might be an important limitation in studies employing predetermined entries, previous studies have demonstrated comparable, and relatively high, compliance in electronic and paper diary studies, when using a random-entry design [66], [67], [68], also employed in the present study. Further, it is possible that utilizing different time lags in the predictive analyses would have led to different results.

The findings have a number of clinical implications. Various psychotherapies operate by means of modifying coping strategies – though often using different methods (for review, see [69]); the response style theory has been found to provide a useful framework for understanding the utility of coping styles. Our findings highlight the importance of therapeutic strategies to ameliorate rumination in bipolar patients, and also the potential value of psychoeducational methods of reducing risk taking in response to incipient manic symptoms. The observation that risk-taking prompted by positive affect leads to a further escalation of affect points to the need to interrupt this cycle during the earliest phase of a hypomanic episode. Existing cognitive behavior therapy strategies which have been shown to be effective already address these issues to some degree [70]. The results regarding adaptive coping are promising as they imply that individuals with severe illness retain some ability to effectively regulate their mood.
